# Cross-sectional study of coronavirus disease 2019 (COVID-19) vaccine uptake among healthcare workers

**DOI:** 10.1017/ash.2022.41

**Published:** 2022-04-11

**Authors:** Rachel K. Russ, Theron J. Schultz, Nicole Kalscheur, James H. Conway, Nasia Safdar, Freddy Caldera, Mary S. Hayney

**Affiliations:** 1 School of Pharmacy, University of Wisconsin–Madison, Madison, Wisconsin; 2 Employee Health Services, UW Health, Madison, Wisconsin; 3 Department of Pediatrics, University of Wisconsin School of Medicine & Public Health, Madison, Wisconsin; 4 Division of Infectious Diseases, Department of Medicine, University of Wisconsin School of Medicine & Public Health, Madison, Wisconsin; 5 Division of Gastroenterology and Hepatology, Department of Medicine, University of Wisconsin School of Medicine & Public Health, 800 University Bay Drive, Madison, Wisconsin

Following widespread availability of the coronavirus disease 2019 (COVID-19) vaccine,^1^ an early survey showed that adults who were younger, Black, with lower education attainment or income, and without health insurance were most likely to decline a COVID-19 vaccine.^2,3^ However, the acceptance of the vaccine among healthcare workers (HCWs) is not well understood. We assessed COVID-19 vaccine acceptance among HCWs to identify patterns to guide vaccine outreach efforts.

## Methods

We conducted a cross-sectional study of HCWs at an academic medical center in a Midwestern mid-sized city who were offered the COVID-19 vaccine. They were categorized into (1) those who submitted a religious, philosophical, or personal choice waiver; (2) those who received vaccine; and (3) those who did not respond. HCW demographics, including social vulnerability index (SVI) were collected on March 29, 2021. SVI considers housing, transportation, social economic status, and race, ethnicity, and language to measure social vulnerability on a scale of 0–1, with a higher number equating to higher vulnerability and greater need for support.^4^ Employee addresses and their corresponding census tract were used to assign SVI.

HCWs who completed the BNT162b2 severe acute respiratory coronavirus virus 2 (SARS-CoV-2) vaccine series, those who submitted a waiver, and those who were offered vaccine but took no action were included in the study. Those who had not received second dose at time of data collection were excluded. The study met the requirements for quality improvement and was deemed exempt from institutional review board review. SPSS version 27 software (IBM, Armonk, NY) was used for ANOVA and χ^2^ with correction for multiple tests, logistic regression, and odds ratios.

## Results

Of the eligible HCWs, 12,146 of 14,989 individuals (81%) were fully vaccinated, 1,429 (9.5%) had an active waiver, and 1,414 (9.4%) individuals took no action following invitation to receive COVID-19 vaccine. Those with a waiver (0.37±0.24; Bonferroni corrected *P* = .007) or who took no action (0.35±0.24; Bonferroni corrected *P* = .006) had a higher SVI compared to those who were vaccinated (0.30±0.21) (Supplementary Table 1). Age was similar among the groups: vaccinated (41±12 years); waiver (39±12 years); no action (40±12 years) (*P* = .35). The most common reasons for a waiver were safety (49.6%), medical (16.7%), and moral reasons (13.6%). Women were more likely to indicate safety as the reason for waiver than other reasons than men with waivers (OR, 1.4; 95% CI, 1.0–1.9) (Supplementary Table 2).

**Fig. 1.**
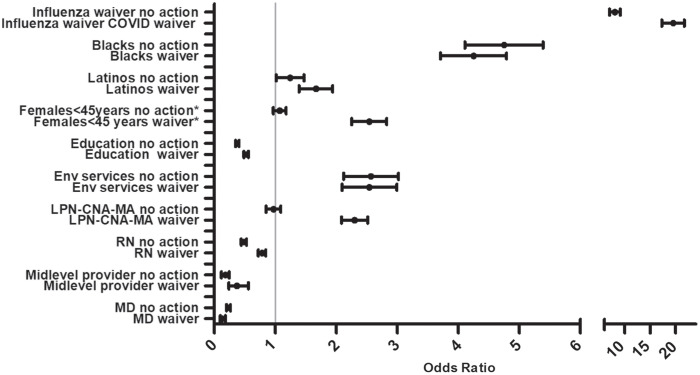
Odds ratios (OR) for COVID-19 vaccine waivers or taking no action following an invitation to receive the vaccine among several healthcare worker groups. Each specified group is compared to all other healthcare workers. The probability of waiver or no action is illustrated by its distance from the line on the graph indicating 1. The odds ratio (OR) with 95% confidence intervals that cross the line have a probability that is similar in the 2 groups (ie, the group named on the y axis compared to all other healthcare workers). Education less than a bachelor’s degree, Black race, Latino ethnicity, higher social vulnerability index (SVI), and having an influenza vaccine waiver had higher ORs for waiver or no action with the COVID-19 vaccine. Physicians, mid-level providers, and nurses (RN) had low ORs. The group with licensed practical nurses, certified nursing assistants, medical assistants (ie, LPN, CNA, and MA) had an increased odds for waiver.

Women of reproductive age (<45 years) were more likely to submit a waiver than men of the same age (OR, 2.5; 95% CI, 2.1–3.0). Those with at least a bachelor’s degree were less likely to have an active waiver or to take no action, whereas Blacks and Latinos were more likely than Whites to have an active waiver or to take no action (Fig. 1).

Physicians, mid-level providers, and registered nurses were less likely to have an active waiver or take no action when compared to all other HCWs. Environmental service technicians, medical assistants, and those with an influenza waiver were all more likely to have an active waiver or take no action when compared to other HCWs (Fig. 1). A logistic regression model included age (β coefficient, 0.015), SVI (β coefficient, −0.83), race and ethnicity (β coefficient, 0.051), and at least bachelor’s degree (β coefficient, 0.69) at *P* < .001.

## Discussion

Our study shows that while most HCWs received COVID-19 vaccines, considerable vaccine hesitancy existed among HCWs who were younger, who were Black or Latino, who were hesitant about an influenza vaccine, and who had less education. These findings mirror early patterns seen within the general population.^3^ This study highlights vaccine hesitancy patterns related to socioeconomic status through SVI, education, and job position. The results of an ongoing survey evaluating COVID-19 vaccine hesitancy among US adults suggest that age and education may be the strongest predictors of vaccine resistance, spanning ethnicities and political preferences.^2^ The SVI observed among HCW who submitted waivers highlight more vulnerable populations among those declining the vaccine.

The National Academies of Sciences, Engineering & Medicine proposed the use of SVI in their consensus report, Framework for Equitable Allocation of COVID-19 Vaccine, in Fall 2020.^5^ This index is calculated for each US Census tract based on 15 social factors and was developed for allocation of resources during a public health emergency. Use of the SVI was encouraged because people of color had higher rates of COVID-19 morbidity and mortality due to the impacts of systemic racism and socioeconomic variables (eg, crowded housing, employment as frontline workers), and they have more severe disease when infected. Even though SVI was used for vaccine allocation, it was also a factor in vaccine hesitancy and waiver.

The study had several limitations. The health history of the HCWs was not known, and a compliance deadline for HCWs to be vaccinated or submit a waiver had not been set allowing delays for individuals acting on their invitation or waiver.

This study underscores the need for focused vaccine outreach and interventions to ensure equitable vaccine access and education to address the safety concerns revealed in this study. We have conducted outreach efforts to specific groups that had lower COVID-19 vaccine uptake, including educational videos and electronic signs. We continue to review declination reasons and to target education and communication toward addressing these concerns. The issue spans beyond COVID-19 vaccinations; similar waiver trends were seen among the same HCW population when evaluating influenza vaccine waivers.^6^ Our analysis gives insight into where those efforts could be directed.

## Supplementary material

For supplementary material accompanying this paper visit https://doi.org/10.1017/ash.2022.41. click here to view supplementary material
