# Supplementary material for: Cross-sectional study of coronavirus disease 2019 (COVID-19) vaccine uptake among healthcare workers
Source: Antimicrob Steward Healthc Epidemiol. 2022 Apr 11;2(1):e61. doi: 10.1017/ash.2022.41 (PMC9726565; doi:10.1017/ash.2022.41)
Supplement: Supplementary file 1 [file ashsup.zip › S2732494X22000419sup001.docx]

| **Supplemental Table 2. Waiver Reasons** | | | |
| --- | --- | --- | --- |
|  | Safety | All other reasons | OR (95%CI) |
| White | 555 (52.3%) | 527 (48.2%) | 0.86 (0.59-1.25) |
| African American | 59 (47.6%) | 65 (52.4%) |  |
|  | | | |
| Females | 604 (50.9%) | 583 (49.1%) | 1.40 (1.04-1.90) |
| Males | 85 (42.5%) | 115 (57.5%) |  |
|  | | | |
| Nurses | 144 (49.5%) | 147 (50.5%) | 1.0 (0.77-1.29) |
| All other job categories | 562 (49.4%) | 576 (50.6%) |  |
|  |  |  |  |
| Environmental services | 19 (37.3%) | 32 (63.7%) | 0.6 (0.33-1.06) |
| All other job categories | 687 (49.9%) | 689 (50.1%) |  |
|  |  |  |  |
| LPN-CNA-MA | 114 (53.8%) | 98 (46.2%) | 1.2 (0.92-1.65) |
| All other job categories | 592 (48.6%) | 625 (51.3%) |  |
|  |  |  |  |
|  | | | |
| Higher education^a^ | 179 (45.5%) | 214 (54.5%) | 1.24 (0.98-1.56) |
| Less education | 527 (71.6%) | 209 (28.4%) |  |
|  | | | |
| Below SVI mean | 366 (49.1%) | 379 (50.9%) | 1.02 (0.83-1.25 |
| Above SVI mean | 340 (49.7%) | 344 (50.3%) |  |
| OR (95% CI) odds ratio with 95% confidence intervals  SVI: social vulnerability index  LPN-CNA-MA: licensed practical nurse, certified nursing assistant, or medical assistant | | | |
| ^a^ Higher education is defined as a bachelor degree, graduate school and above, master degree, doctorate, or postdoctorate degree. Less education is defined as < secondary basic cycle, high school grad, college level, 2-year junior college, associate degree, other education level, technical/industrial Institute, or blank. | | | |
