# Supplementary material for: Cross-sectional study of coronavirus disease 2019 (COVID-19) vaccine uptake among healthcare workers
Source: Antimicrob Steward Healthc Epidemiol. 2022 Apr 11;2(1):e61. doi: 10.1017/ash.2022.41 (PMC9726565; doi:10.1017/ash.2022.41)
Supplement: Supplementary file 1 [file ashsup.zip › S2732494X22000419sup002.docx]

| **Supplemental Table 1. Healthcare Worker Demographics and Vaccine Uptake Odds** | | | | | | | | | |
| --- | --- | --- | --- | --- | --- | --- | --- | --- | --- |
| Variable | Vaccinated  No. (%) | | Active waiver  No. (%) | | Corrected p value^a^ compared to vaccinated | No action^b^  No. (%) | | Corrected p value compared to vaccinated | |
| **N** | 12146 (81.1%) | | 1429 (9.5%) | |  | 1414 (9.4%) | |  | |
| **Age** | | | | | | | | | |
| Age, years, mean ± SD | 41.3 ± 12.2 | | 38.5 ± 11.5 | | 0.34 | 40.3 ± 12.3 | | 0.35 | |
| OR (95% CI) <49 years^c^ | Reference | | 1.5 (1.3-1.7) | |  | 1.2 (1.1-1.4) | |  | |
| **SVI** | | | | | | | | | |
| Mean ± SD | 0.30 ± 0.21 | | 0.37 ± 0.24 | | 0.007 | 0.34 ± 0.24 | | 0.006 | |
| OR (95% CI) >mean^d^ | Reference | | 1.6 (1.4-1.8) | |  | 1.3 (1.2-1.6) | |  | |
| **Sex** |  | |  | |  |  | |  | |
| Male | 3231 (86.4%) | | 200 (5.4%) | | <0.001 | 307 (8.2%) | | <0.001 | |
| Female | 8582 (81.6%) | | 1,187 (11.1%) | |  | 884 (8.3%) | |  |  |
| Unknown | 333 (55.7%) | | 42 (7.0%) | |  | 223 (37.2%) | |  |  |
| OR (95% CI) female | Reference | | 2.2 (1.9-2.6) | |  | 1.1 (0.95-1.2) | |  | |
| **Race/ Ethnicity** | | | | | | | | | |
| White | 9718 (83.5%) | | 1,082 (9.3%) | | <0.001 | 845 (7.3%) | | <0.001 | |
| African American | 266 (53.4%) | | 124 (24.9%) | |  | 108 (21.7%) | |  |  |
| Latino | 309 (75.4%) | | 58 (14.1%) | |  | 43 (10.5%) | |  |  |
| Other^e^ | 1,853 (76.1%) | | 165 (6.8%) | |  | 418 (17.2%) | |  |  |
| OR (95% CI) White compared to all other categories | Reference | | 0.78 (0.68-0.89) | |  | 0.37 (0.33-0.42) | |  | |
| **Education**^f^ |  | |  | |  |  | |  | |
| Higher education | 5117 (42.1%) | | 393 (27.5%) | | <0.001 | 303 (21.4%) | | <0.001 | |
| Less education | 7029 (57.9%) | | 1,036 (72.5%) | |  | 1111 (78.6%) | |  |  |
| OR(95%CI) at least Bachelor degree | Reference | | 0.52 (0.46-0.59 | |  | 0.28 (0.24-0.33) | |  | |
| **Job Title**^g^ | | | | | | | | | |
| Physician | 2228 (95.2%) | | 41 (1.8%) | |  | 71 (3.0%) | |  | |
| OR(95% CI) compared to all other positions | Reference | | 0.13 (0.10-0.18) | |  | 0.23 (0.18-0.29) | |  | |
| Mid-level provider^h^ | 521 (94.0%) | | 23 (4.2%) | |  | 10 (1.8%) | |  | |
| OR(95% CI) compared to all other positions | Reference | | 0.27 (0.24-0.56) | |  | 0.16 (0.08-0.30) | |  | |
| RN | 3002 (86.2%) | | 291 (8.4%) | |  | 191 (5.5%) | |  | |
| OR(95% CI) compared to all other positions | Reference | | 0.78 (0.68-0.89) | |  | 0.48 (0.41-0.56) | |  | |
| LPN-CNA-MA | 862 (73.7%) | | 212 (18.1%) | |  | 96 (8.2%) | |  | |
| OR(95% CI) compared to all other positions | Reference | | 2.28 (1.94-2.68) | |  | 0.95 (0.77-1.19) | |  | |
| Environmental  Services | 187 (63.6%) | | 53 (18.0%) | |  | 54 (18.4%) | |  | |
| OR(95% CI) compared to all other positions | Reference | | 2.46 (1.81-3.36) | |  | 2.54 (1.81-3.36) | |  | |
| **Influenza vaccine status** | | | | | | | | | |
| No waiver | 11,972 (83.3%) | | 1,116 (7.8%) | | <0.001 | 1,268 (8.8%) | | <0.001 | |
| Waiver | 174 (27.5%) | | 313 (49.4%) | |  | 146 (23.1%) | |  |  |
| OR(95% CI) no influenza vaccine | Reference | | 19.3 (15.9-23.5) | |  | 7.9 (6.3-10.0) | |  | |
| Incidence of vaccine predictors among selected job categories | | | | | | | | | |
|  | | Influenza Waiver Rate | | Female | | | White | | Black or African American |
| Physicians (n=2381) | | 28 (1.2%) | | 1066 (44.8%) | | | 1701 (71.4%) | | 46 (1.9%) |
| Nurses (n=3654) | | 95 (2.6%) | | 3173 (86.8%) | | | 3152 (86.3%) | | 36 (0.1%) |
| Environmental services (n=320) | | 41 (12.8%) | | 159 (49.7%) | | | 109 (34%) | | 111 (34.7%) |
| LPN-CNA-MA (n=1202) | | 44 (3.6%) | | 1039 (86.4%) | | | 836 (69.6%) | | 98 (8.2%) |
| SD: Standard deviation  OR (95% CI) odds ratio with 95% confidence intervals  SVI: social vulnerability index  LPN-CNA-MA: licensed practical nurse, certified nursing assistant, or medical assistant | | | | | | | | | |
| ^a^ Bonferroni post-hoc test following ANOVA or chi square test.  ^b^ No action includes those who did not respond to the invitation to receive COVID-19 vaccine  ^c^ OR calculated using age 19-49 years and 50 years and older as risk groups.  ^d^ OR calculated using less than and greater than mean SVI (0.31) for entire cohort  ^e^ Other race includes American Indian or Alaska Native, Asian, Native Hawaiian or other Pacific Islander, two or more ethnic groups, not specified, and blank responses  ^f^ Higher education is defined as a bachelor degree, graduate school and above, master degree, doctorate, or postdoctorate degree. Less education is defined as < secondary basic cycle, high school grad, college level, 2-year junior college, associate degree, other education level, technical/industrial Institute, or blank.  ^g^ Not all the numerous job categories are listed; Chi square test not done as the entire study cohort is not included in the table.  ^h^ Mid-level provider nurse practitioner, physician assistant, or midwife | | | | | | | | | |
